# THE COMPREHENSIVE 360° MODEL OF RESEARCH: ITS FOUNDATIONS AND EXEMPLARY APPLICATION IN SPINAL CORD INJURY

**DOI:** 10.2340/jrm.v57.42019

**Published:** 2025-02-12

**Authors:** Sara RUBINELLI, Jerome BICKENBACH, James W. MIDDLETON, Ian D. CAMERON, Carla SABARIEGO, Gerold STUCKI

**Affiliations:** 1 Swiss Paraplegic Research (SPF), Nottwil, Switzerland; 2 Faculty of Health Sciences and Medicine, University of Lucerne, Lucerne, Switzerland; 3 Center for Rehabilitation in Global Health Systems, University of Lucerne, Lucerne, Switzerland; 4 Kolling Institute, Faculty of Medicine and Health, The University of Sydney, Sydney, NSW, Australia; 5 John Walsh Centre Rehabilitation Research, Northern Sydney Local Health District, St Leonards, Sydney, NSW, Australia

**Keywords:** health status indicators, functioning, interdisciplinary research, patient-centred care, rehabilitation, spinal cord injuries, health policy, health systems

## Abstract

**Objective:**

This paper introduces the “360° Model of Research”, a novel framework designed to prioritize functioning as a primary health indicator, particularly for individuals living with health conditions. The goal is to promote this model as a comprehensive, person-centred, and interdisciplinary approach to health research, moving beyond the traditional siloed and disease-based methodologies.

**Methods:**

The paper is conceptual, integrating existing scientific literature and theoretical frameworks to build the 360° Model. It draws on interdisciplinary perspectives from rehabilitation, social sciences, health services research, and ethics, among others, to create a unified health science focused on optimizing functioning.

**Results:**

The 360° Model emphasizes functioning as a key health indicator, combining biological health with lived experience. The Model provides a reference framework for data collection, but does not presume any particular explanatory theory about how lived experience is created or interpreted by the individual. It has been successfully applied in the Swiss Spinal Cord Injury Cohort Study (SwiSCI), demonstrating its value in addressing the comprehensive needs of individuals with spinal cord injury through interdisciplinary collaboration and a learning health system theory.

**Conclusion:**

The 360° Model offers a transformative approach, highlighting the importance of functioning in health research. It provides a robust foundation for a new interdisciplinary field of Human Functioning Sciences, aimed at optimizing health and well-being across diverse populations.

## LAY ABSTRACT

This paper presents the “360° Model of Research”, a novel approach to studying health that focuses on how well people function in their daily lives, especially when living with health conditions. Instead of just looking at disease symptoms or how long people live, this model puts the spotlight on functioning: how health conditions affect a person’s ability to live their life fully. The model combines insights from different fields, as in the health sciences and social sciences, to create a complete picture of health. It has already been used successfully in research on spinal cord injury in Switzerland, helping to improve care and support for people with these injuries. The 360° Model is a step towards a new way of thinking about health, one that aims to improve the functioning of everyone, no matter their health challenges.

Countries and their health systems worldwide face the dual challenges of an ageing population and a steady increase in chronic health conditions (1–3). Together these trends will place a considerable strain on health and social systems. While the need to address the essential health goals of reducing mortality and morbidity will continue, ageing and chronic conditions will increase the prevalence of limitations in functioning and disability in the population (4–7). The societal responsibility to take appropriate actions to address these ever-increasing functioning needs is entailed by the fundamental societal obligations, stated in documents such as the International Covenant on Economic, Social and Cultural Rights (8), to enjoy the highest attainable level of physical and mental health. Although how the societal response should be provided for, financed, and technically supported may be a contentious issue, it is uncontroversial that society is responsible for creating the basic infrastructures and providing the financial and human resources necessary to respond to the health and functioning needs of the population.

This societal commitment to population health must be grounded in the health sciences to provide the evidence base for healthcare practice and policy. At the core of health research is understanding the lived experience of individuals with health conditions. The societal response must, therefore, be shaped to respond to the actual health needs of individuals. Increasingly, those needs have been expressed in terms not merely of decreasing mortality and morbidity, but of optimizing functioning (9) – the core concept underlying the World Health Organization’s International Classification of Functioning, Disability, and Health (ICF) (10). By focusing on functioning as the third indicator of health, health research can both address the global population and epidemiological challenges, and more effectively shape the societal response to meet people’s health needs.

However, research that can comprehensively capture the individual’s lived experience of health requires more than creating new research designs or methodologies. To be truly effective, a new and robust research model must be described that fully capture the actual lived experience of health. We propose that a new model of health research should be constructed on 3 essential pillars that together create a comprehensive, effective, and socially responsible approach to health research.

*A foundational perspective and an operational definition of health:* This first pillar is crucial because it requires a clear, consistent understanding of what health means in the context of research. A foundational perspective on health provides the conceptual clarity needed to guide research and ensure that all studies are aligned with a common understanding of health. Operationalizing this definition directly in terms of the ICF’s biopsychosocial model allows researchers to translate abstract concepts into measurable outcomes, making it possible to assess the impact of various factors on health in a systematic and comparable way. This clarity is essential for producing research that is meaningful, reliable, and applicable across different contexts.

*An interdisciplinary scientific approach that integrates diverse perspectives on health:* Health is a multifaceted phenomenon influenced by biological, psychological, social, and environmental factors (11). To fully understand and address health issues, it is necessary to draw on knowledge from multiple disciplines. An interdisciplinary approach breaks down the gap that often exists between these fields, fostering collaboration and the integration of diverse perspectives. This comprehensive view enables researchers to capture the complexity of health and to develop more comprehensive and effective interventions.

*A normative framework that grounds research in society’s responsibility to address population health needs:* The third pillar emphasizes the ethical and social dimensions of health research. Health research does not exist in a vacuum; it is conducted within the context of societal values and responsibilities. A normative framework provides the ethical foundation for health research, ensuring that it aligns with society’s commitment to improving population health. This framework guides the prioritization of research questions, the allocation of resources, and the application of research findings in ways that are just and equitable. It also underscores the moral obligation of society to respond to the health needs of its population, particularly those who are most vulnerable.

The objective of this paper is to introduce, advocate for, and exemplify what we call the “360° Model of Research” – a framework specifically designed to study functioning in order to capture the complex realities of living with 1 or more health conditions, and which provides an evidence-based platform for shaping the response to health needs that it is society’s responsibility to provide. The model is inherently both person-centred and comprehensive. The person-centred approach ensures that research places the individual at the core of inquiry, with a focus on their unique experiences, needs, and preferences as they manage their health conditions. Simultaneously, the model’s comprehensiveness is essential for encompassing the full spectrum of factors that influence functioning and, it has been argued, well-being itself (12).

Specifically, our objectives are to provide a workable description of “|functioning” as the conceptual foundation for comprehensive health research. Our next objective is to explore the potential of a functioning-based unified field of health science, emphasizing the integration of interdisciplinary perspectives to create a comprehensive understanding of health. The third objective is to highlight the normative dimension and the importance of translating research into real-world applications. The paper then transitions from theoretical foundations to practical applications, presenting as the fourth objective a case study of over a decade of research conducted by Swiss Paraplegic Research, an institute focused on spinal cord injury (SCI). This case study exemplifies the effectiveness of the 360° Model in addressing the comprehensive needs of individuals living with SCI. Finally, the paper concludes by advocating for the broader adoption of the 360° Model across various health conditions, highlighting its potential to significantly enhance societal efforts to optimize the functioning and well-being of diverse patient populations.

## RESULTS AND DISCUSSION

### Functioning, the conceptual foundation for comprehensive health research

In this section, we demonstrate why “functioning” is the most appropriate conceptual foundation for the 360° Model of Research.

In the World Health Organization’s (WHO) International Classification of Functioning, Disability and Health (ICF), (9) the term “functioning” is formally defined as the set of body functions and structures and the totality of human activities, from the very basic and simple (walking, seeing, hearing) to the more complex (reading, activities of daily living) to the highly complex, socially constructed areas of human life in which people participate (having a family, working, engaging in education, participating in community life).

However, the ICF is more than just a classification system. It embodies a comprehensive biopsychosocial model where each aspect of body functions, structures, activities, and social participation is deeply contextualized by environmental factors – ranging from climate and the built environment to the attitudes and beliefs of others, as well as broader social and political systems. Additionally, personal factors, such as gender, place of birth, coping mechanisms, and self-efficacy, also play a crucial role. Together, these components create a language that accurately describes an individual’s health situation in the real world.

Conceptually, functioning is the result of dynamic interactions between our biomedical health and the various features of the world in which we live. It encompasses how we function, behave, and act within our environment, essentially capturing the lived experience of health (13). Moreover, the ICF understanding of functioning and disability is grounded in 3 core principles (14): the first is that disability is a universal human experience, a fact of human life, not the mark of a demographic minority. Second, disability is aetiologically neutral, in the sense that a decrement in functioning cannot be solely predicated on the presence or characteristics of a specific health condition, because both the existence and nature of disability also depends on the person’s environment. Finally, the biopsychosocial model entails that disability lies on a continuum from no disability (or full functioning) to complete disability. Taken together, these principles determine the range and sources of data describing the experience of describing, without providing a theory explaining how the person–environment interaction operates. This last point is crucial for the current discussion because, from the earliest conception of the ICF biopsychosocial model, the aim was to provide a conceptual framework that was theory-neutral, in the sense of being compatible with a wide range of specific, theoretical accounts of how the disability experience arises. This biopsychosocial model – defined by the 3 core principles and theory-neutrality – is the basis for a comprehensive view of health as lived experience and the 360° Model of Research we are proposing here.

The ICF concept of functioning has been widely adopted in rehabilitation research and clinical practice, and it serves as a cornerstone for other models, such as the Canadian Model of Occupational Performance (15), the Person-Environment-Tool Model (16), and the Matching Person and Technology Model (17). Among these, the ICF stands out as the only international classification system that provides a universal framework for functioning, applicable across various contexts and not limited to a specific purpose. It is also such frameworks that are committed to theory-neutrality, as the concept of functioning does not depend on an explanatory or ontological account of how functioning is actually shaped by the interaction between person and environment, but rather is open to a wide range of such theories.

For the 360° Model, functioning is not just a measure of health; it is a comprehensive operationalization that bridges biological health – the intrinsic state of body functions and structures – with lived health – the actual performance of activities in interaction with one’s environment, other health conditions, and personal factors (18). This dual integration of biological and lived health underscores the concept’s suitability as the foundation for a new, comprehensive approach to health sciences. This approach is a framework, not an explanatory or ontological theory; it provides us with a reference framework for data collection from the perspective of the person, without offering a theory concerning that lived experience.

The ICF biopsychosocial model provides a robust reference framework for data collection as well as a vocabulary for describing and coding aspects of the disability experience, in terms of both intrinsic bodily states, activities and participation, and the range of services, entitlements, resources, and policies that form the societal response to health needs. In healthcare, these responses are typically broadly categorized as the health strategies of prevention, health promotion, curative treatment, rehabilitation, and palliation (19). Broadly speaking, any societal resource, policy, or action that directly or indirectly influences environmental risk factors or social determinants of health is part of the overall societal response to individual and population health (20). This broad applicability reinforces why functioning is the appropriate and comprehensive foundation for the 360° Model of Research.

The need for such a comprehensive approach to health and its social responses calls upon the resources of a full range of health science disciplines, including biomedical, clinical, epidemiological, and socio-humanistic sciences. This interdisciplinary integration is crucial for a thorough exploration of the lived experience of health. The ICF serves as the “glue” that holds together these diverse scientific perspectives and theories, making the 360° approach not only feasible but also uniquely powerful in advancing health research. This sets the stage for the next section, where we explore the prospect of a unified field of health sciences under the concept of functioning, promoting true interdisciplinarity in health research.

### The prospect of a functioning-based unified field of health science

Typically, health research has been driven by the immediate needs of individuals suffering from specific health conditions. This targeted approach is often shaped by disease prevalence and burden, emphasizing the perceived importance of addressing particular diagnoses. As a result, scientific disciplines have tended to specialize in studying only these diagnosed conditions, often overlooking the broader picture of an individual’s overall health state or their comprehensive experience of living with the health condition. Consequently, health research – and the scientific journals that disseminate its findings – has become overwhelmingly compartmentalized, focusing on specific diseases, injuries, syndromes, or impairments. This segmentation reflects a fragmented view of health, primarily concerned with biomedical questions such as disease processes, genetics, or standard epidemiological outcomes like mortality and morbidity. Such a focus fails to capture a comprehensive understanding of health and well-being.

Over the past decades, health sciences research has expanded significantly, drawing on contributions from a wide array of scientific disciplines, each rooted in different intellectual traditions (21). Discipline-specific research has undoubtedly benefited from the insights of natural sciences and has gradually embraced perspectives from the social sciences, engineering, and even the humanities. Each discipline has introduced methodological innovations and research designs that have enriched the overall body of knowledge. However, despite these advancements, the diversification of research into specialized fields has perpetuated a pattern of segmented and siloed research. This, in turn, has created professional and conceptual barriers to collaboration among researchers, who often align themselves with distinct disciplinary cultures. The challenge, then, is to transform health sciences – comprising multiple disciplines with varying perspectives – into a coherent and integrated field of study.

The 360° Model of health research offers a promising solution to this challenge. By focusing on the comprehensive, lived experience of individuals, this model promotes engagement and interdisciplinary collaboration, breaking down the silos that hinder integration. It encourages a unified understanding of health that draws on insights and findings from various disciplines. The true potential of the 360° Model lies in its ability to foster emergent novelty and innovation. By integrating the results of different scientific disciplines, the model generates deeper insights into health than what could be achieved through isolated, discipline-specific research. The 360° Model not only bridges gaps between different scientific fields but also enriches our understanding of health by viewing it through a multifaceted lens. The data framework that the Model applies is theory-neutral in the important sense that it is compatible with a wide range of theoretical explanation from difference disciplines. This approach in short paves the way for more effective, comprehensive, and nuanced health research, ultimately leading to better health outcomes and person-centred care.

We have previously argued that a conceptual model of health sciences, as an integrated field of study, is rooted in the fundamental interaction between an individual’s health and society’s organized response to health needs, which is delivered through its health and related societal structures (21). The ICF’s notion of functioning shifts the focus from the health problems themselves to the individual’s lived experience of health. Functioning provides the integrative tissue that connects the individual and society and serves as the platform for interdisciplinary research (22). As the lived experience of health is inevitably shaped by the complete environment – physical, human, and social – in which one lives, understanding this diverse environment is essential. This necessity brings together a wide variety of scientific disciplines, all of which contribute to our understanding of health at the individual, system, and population levels.

Ultimately, the goal is to use health sciences as an integrated field of study to provide the evidence base for society’s response to health needs. This integrated approach aims to explain and predict health phenomena and generate innovations in interventions and other societal responses to health needs, all with the ultimate goal of optimizing individual and population functioning.

A more comprehensive description of a unified field of health sciences would require a typology of methodologies and research designs that brings together the distinct explanatory powers of conceptual analysis, as well as qualitative, quantitative, and mixed methodologies. Our point here is to illustrate how the 360° Model of Research both presupposes and fosters such a comprehensive and integrated view of health sciences – a truly interdisciplinary health science. At its core is the understanding that what matters most about our health is how it shapes our lives, both as individuals and as societies that bear the responsibility to optimize that experience.

### The normative foundation and implementation of the 360° Model of Health Research

Building on the interdisciplinary and functioning-based foundation of the 360° Model, there is a third crucial aspect that must be addressed: the normative. It is this dimension of the 360° Model we are proposing that moves beyond the original ICF biopsychosocial model, defined by the 3 principles already mentioned of universality, aetiological neutrality, and continuity of functioning. Implicit in the implementation of a framework for research is the recognition that the scientific outcomes generated by this model are not produced in isolation; they are deeply embedded within societal contexts and are fundamentally shaped by societal institutions. Central to this complex process are implicit or explicit propositions concerning the nature and extent of society’s responsibility to address the health needs of both individuals and populations. As scientific and technological advancements have expanded the possibilities for improving health, the scope of societal responsibility has also evolved. However, despite this evolution, the core consensus remains: society has an obligation to ensure the health and well-being of its citizens.

Scientific research, particularly in health, is a significant social investment. It is not merely an academic exercise but a serious endeavour that must justify its costs by demonstrating its value to the public good. Health research, in particular, is grounded in the moral imperative for societies to respond effectively to the health needs of their populations. The research must lead to practical applications that enhance public health outcomes. Ultimately, the specific ways in which societies choose to respond to health needs are shaped by political negotiations and consensus on normative values. While the health sciences themselves may not dictate these values, they provide the critical evidence base that informs and supports societal decisions on health policy and practice.

The final pillar of the 360° Model of Health Research is, therefore, a normative framework – an agreed-upon scaffold of public values that guide the direction and priorities of health research. This framework ensures that the research conducted aligns with society’s responsibility to address health needs and that it leads to actionable outcomes. It helps to identify and prioritize research questions based on societal needs and ethical considerations, making sure that the research conducted is not only scientifically robust but also socially relevant.

This normative framework also carries an inherent obligation to translate research findings into real-world applications – a process known as implementation. Implementation involves a multi-faceted strategy that brings scientific results and innovations into practice across various levels of society. At the macro level, this might involve changes in health policy or systems; at the meso level, it could mean improvements in service quality management or financing; and at the micro level, it might influence individual clinical practices and patient-reported outcome measurement.

To sustain progress and ensure continuous improvement the implementation process often follows a cyclical pattern. This cycle includes identifying research issues, developing and testing potential solutions, implementing these solutions, monitoring and evaluating their impact and sustainability, and then identifying new issues for further research. One of the most effective approaches to this cyclical implementation is the Learning Health System, conceptualized in a 2006 workshop organized by the US Institute of Medicine (now the National Academy of Medicine [NAM]) (23) and which is particularly well suited to the functioning-based focus of the 360° Model. This approach emphasizes the ongoing process of learning and adaptation within health systems, ensuring that research continually informs practice and policy in a dynamic, responsive manner.

We now turn to the SwiSCI Cohort Study to provide a concrete example of how the 360° Model is applied in practice, demonstrating its potential to enhance societal efforts in optimizing health and well-being.

### Applying the 360° Model of Research: the SwiSCI Cohort Study

Spinal Cord Injury (SCI) represents a life-altering event that profoundly impacts an individual’s health and functioning. Damage to the spinal cord can disrupt all bodily functions below the level of the lesion, predisposing to a wide range of secondary health conditions such as autonomic dysfunctions, pressure injuries, recurrent urinary infections, chronic pain, and spasticity. Severe or recurrent health conditions can interfere with nearly every aspect of a person’s life and continue to do so with the additive effects of ageing with disability throughout their lifespan. Given the relatively low prevalence of SCI, comprehensive, long-term cohort studies have only recently begun to provide the data necessary to deepen our understanding of the lived experience of those with SCI.

The SwiSCI (Swiss Spinal Cord Injury Cohort Study) was initiated with the primary goal of gathering fundamental epidemiological data on SCI in Switzerland, including incidence, prevalence, mortality, and morbidity trends over time (24). However, from its inception in 2010, the SwiSCI study expanded its scope beyond traditional epidemiological objectives. It has emphasized understanding the lived experience of SCI, focusing on the various dimensions of functioning for individuals undergoing first rehabilitation as well as persons living in the community. This approach aligns closely with the principles of the 360° Model of Research and a framework for collecting data concerning the lived experience of SCI that makes the SwiSCI study a prime example of how this model can be applied in practice. The model’s flexibility is further demonstrated by its capacity to incorporate qualitative data, acknowledging that in-depth understanding of lived experiences often requires approaches beyond quantitative measures (25).

**Fig. 1:**
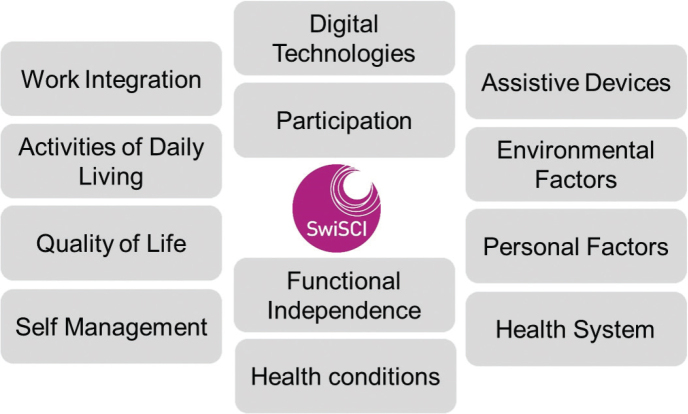
SwiSCI data framework embodying the 360° research approach

The data collected through the 3 pathways of the SwiSCI study offer a rich, multifaceted view of the impact of SCI on individuals’ lives. These insights not only enhance our understanding of SCI but also demonstrate the practical value of the 360° Model in capturing the full spectrum of health experiences and determinants related to a specific condition. The biopsychosocial ICF model (9) is the reference framework of SwiSCI. To ensure that all aspects relevant to the lived experience of persons with SCI are covered by SwiSCI, selections of ICF categories encompassing the components of functioning and environmental factors most relevant to SCI were used, namely the ICF Core Sets for SCI in the long term (26) and in the early post-acute context (27). Researchers used the ICF Core Sets for spinal cord injury to identify and select relevant categories of body function and structure for the SwiSCI study (28). To ensure that SwiSCI data are comparable to other disease- or condition-specific cohorts, the SCI-specific Core Sets were complemented with categories of a generic Core Set, known as the ICF-Generic 30 (29) This approach leads to a cohort study that includes modules covering a range of elements relevant to the lived experience, from the structure of the health system to self-management strategies used by persons with SCI in daily life. Fig. 1 shows topics covered by the SwiSCI Cohort Study, reflecting its commitment to the 360° approach centred on functioning. While this approach might resemble the breadth of ageing cohorts, it is much broader than usual condition-specific cohorts.

The SwiSCI study, a concrete example of the 360° Model in action, uses the International Classification of Functioning, Disability and Health (ICF) as a framework to gather comprehensive data on the lived experience of spinal cord injury. This includes detailed considerations of functioning, participation, and environmental factors (25, 30, 31).

The success of the SwiSCI study in comprehensively capturing the lived experience of SCI paved the way for the development of the Swiss Learning Health System for Spinal Cord Injury (SLHS-SCI) (32). The SLHS-SCI was designed to expand the scope of the SwiSCI study by not only continuing to collect extensive data on SCI but also embedding these data within a broader framework that includes normative guidelines and implementation strategies.

The SLHS-SCI serves as an integrative research platform with the goal of translating research into actionable outcomes that directly benefit individuals living with SCI. It achieves this by structuring its approach around 2 key components:

*A normative scaffolding*: This framework is grounded in a consensus of values that are critically relevant to the lived experience of individuals with SCI. The scaffolding draws on internationally recognized standards and agreements, including the WHO-ISCoS document International Perspectives on SCI (IPSCI) (33, 34), the World Health Assembly Resolution on Strengthening Rehabilitation in Health Systems (35), and the human rights principles and requirements enshrined in the United Nations’ Convention on the Rights of Persons with Disabilities (36). These documents provide the ethical and social foundations that guide the SLHS-SCI’s research initiatives, ensuring that they align with global best practices and uphold the fundamental rights of people with disabilities.

*The Theory of Change framework*: The Theory of Change refers to a comprehensive framework that outlines how and why a desired change is expected to happen in a particular context. In the case of the SLHS-SCI, this theory is realized through the adoption of the Learning Health System approach within the SwiSCI study. This approach is characterized by its dynamic, cyclical process, where health systems continuously identify emerging issues, develop and test evidence-based responses, implement these responses, monitor the outcomes, and then refine and adapt policies and practices based on what is learned. This perpetual loop of learning and improvement allows the SLHS-SCI to effectively translate the vast amounts of data on the lived experience of SCI into practical, evidence-based policies and interventions that enhance health outcomes.

Through the integration of these components, the SLHS-SCI ensures that its research not only contributes to academic knowledge but also results in meaningful improvements in the care and well-being for individuals with SCI. This system exemplifies the full potential of the 360° Model of Research, moving beyond data collection and analysis to the real-world application of research findings in ways that make a tangible difference in people’s lives.

### Expanding the application of the 360° Health Research Model

The 360° Model of health research is designed to be versatile, not limited to conditions with severe, life-altering impacts. While the SwiSCI Cohort Study focuses on SCI – a condition requiring a comprehensive, multifaceted approach due to its profound and wide-ranging effects on the body and life of the individual – the principles of the 360° Model are applicable to a broad spectrum of health conditions.

The SwiSCI study’s success lies in its ability to deeply explore the lifelong trajectory of SCI, considering its dynamic effects on various body systems, the potential for secondary conditions, and its extensive impact on daily activities and social participation. By using an interdisciplinary approach centred on the concept of functioning, the 360° Model provided a detailed, realistic, and person-centred view of what it means to live with SCI. This comprehensive understanding was made possible by integrating diverse scientific perspectives, addressing both the impairments caused by SCI and the role of environmental factors – from assistive technologies to social support systems and policy frameworks – in shaping the lived experience of those affected.

However, the strength of the 360° Model is not confined to SCI. The methodology and principles applied in the SwiSCI Cohort Study can be adapted to other significant health conditions such as stroke, diabetes, or dementia. Each of these conditions is experienced holistically by individuals, affecting multiple aspects of their lives and requiring a comprehensive approach to understand and address their needs fully. The 360° Model’s focus on functioning, rather than the traditional disease outcomes of mortality or morbidity, makes it particularly well suited to these chronic conditions.

To effectively extend the 360° Model to other conditions, the approach would involve creating functioning-based cohorts – groups of individuals characterized not by their specific health conditions, but by their functioning needs. This shift in focus ensures that research addresses the full scope of an individual’s health experience, leading to more meaningful and actionable insights. The process begins by gathering diverse scientific perspectives relevant to the individual’s health experience. These perspectives are then synthesized through the lens of functioning, constructing a comprehensive view of what it means to live with the health condition in question. Furthermore, the 360° Model, guided by socially agreed-upon values regarding society’s responsibility to meet the health-related needs of its people, inherently includes a commitment to implementation. This ensures that all research findings are not only theoretical but are practically applied to improve real-world health outcomes, enhancing the well-being of those affected by various health conditions.

### Conclusion

The 360° Model of Research represents a transformative approach to health sciences, one that is deeply rooted in a comprehensive understanding of the lived experience of health. By prioritizing functioning as a primary health indicator and integrating interdisciplinary perspectives, this model not only addresses the complex realities faced by individuals living with health conditions but also underscores the societal responsibility to meet these needs in a comprehensive and person-centred manner.

The success of the SwiSCI Cohort Study and the subsequent development of the SLHS-SCI illustrate the model’s potential to generate meaningful, actionable insights that extend beyond academic knowledge to make a tangible difference in people’s lives. These initiatives demonstrate that the 360° Model can be effectively applied to other health conditions, offering a versatile and adaptable framework that addresses the full spectrum of the health experience.

Crucially, the 360° Model of Research does more than address current health research needs; it also lays the essential groundwork for establishing a new interdisciplinary field of study: *Human Functioning Sciences*. This emerging field aims to bring together diverse scientific disciplines to explore and understand human functioning in all its complexity. Human Functioning Sciences would systematically study the interplay between biological, psychological, social, and environmental factors that influence how individuals function in their daily lives, especially when living with health conditions. It would build on the foundational concepts of the 360° Model by focusing on the dynamic interactions between a person’s intrinsic health state, their environment, and their lived experience. This field would not only investigate how people manage and adapt to health challenges but also how societal structures, technologies, and policies can be optimized to support better functioning and well-being for all individuals.

**Table I:** The normative “scaffold” that guides research and implementation in the SwiSCI Cohort Study

WHO Resolution on “Strengthening rehabilitation in health systems”	IPSCI recommendations	United Nations Convention on the Rights of Persons with Disabilities
		**Article 1 – Purpose** The purpose of the present Convention is to promote, protect, and ensure the full and equal enjoyment of all human rights and fundamental freedoms by all persons with disabilities, and to promote respect for their inherent dignity Persons with disabilities include those who have long-term physical, mental, intellectual, or sensory impairments which in interaction with various barriers may hinder their full and effective participation in society on an equal basis with others
1. To **raise awareness of and build national commitment for rehabilitation**, including for assistive technology, and strengthen planning for rehabilitation	Improve health sector response to SCI Actions: include SCI topics in the training curriculum for medical and allied health professionals to raise awareness about SCI and promote SCI research; promote access to specialist training to ensure an adequate supply of suitably trained health professionals; engage with policy-makers and other key stakeholders to promote the implementation of the Report’s recommendations	**Article 8 – Awareness-raising** 1. States Parties undertake to adopt immediate, effective and appropriate measures: To raise awareness throughout society, including at the family level, regarding persons with disabilities, and to foster respect for the rights and dignity of persons with disabilities; To combat stereotypes, prejudices, and harmful practices relating to persons with disabilities, including those based on sex and age, in all areas of life; To promote awareness of the capabilities and contributions of persons with disabilities
2. To incorporate appropriate ways to **strengthen financing mechanisms** for rehabilitation services	Improve health sector response to SCI Actions: Governments can ensure that appropriate insurance schemes exist that can protect people against the costs of injury	**Article 25 – Health** States Parties recognize that persons with disabilities have the right to the enjoyment of the highest attainable standard of health without discrimination on the basis of disability. States Parties shall take all appropriate measures to ensure access for persons with disabilities to health services that are gender-sensitive, including health-related rehabilitation. In particular, States Parties shall: Provide persons with disabilities with the same range, quality, and standard of free or affordable health care and programmes as provided to other persons, including in the area of sexual and reproductive health and population-based public health programmes; Provide those health services needed by persons with disabilities specifically because of their disabilities, including early identification and intervention as appropriate, and services designed to minimize and prevent further disabilities, including among children and older persons; Provide these health services as close as possible to people’s own communities, including in rural areas; Require health professionals to provide care of the same quality to persons with disabilities as to others, including on the basis of free and informed consent by, inter alia, raising awareness of the human rights, dignity, autonomy, and needs of persons with disabilities through training and the promulgation of ethical standards for public and private health care; Prohibit discrimination against persons with disabilities in the provision of health insurance, and life insurance where such insurance is permitted by national law, which shall be provided in a fair and reasonable manner; Prevent discriminatory denial of health care or health services or food and fluids on the basis of disability
3. To **expand rehabilitation to all levels of health, from primary to tertiary,** and to ensure the **availability and affordability** of quality and timely rehabilitation services, **accessible** and usable for persons with disabilities, and to **develop community-based rehabilitation strategies**	Improve health sector response to SCI (Support employment and self-employment) (Empower people with SCI and their families) Actions: Governments can improve provision of health, rehabilitation and support services for people with SCI; develop community-based rehabilitation initiatives in resource-poor and remote settings; help strengthen existing (and support the establishment of new) resource-sensitive, appropriate, and timely SCI healthcare services	**Article 26 – Habilitation and rehabilitation** 1. States Parties shall take effective and appropriate measures, including through peer support, to enable persons with disabilities to attain and maintain maximum independence, full physical, mental, social, and vocational ability, and full inclusion and participation in all aspects of life. To that end, States Parties shall organize, strengthen and extend comprehensive habilitation and rehabilitation services and programmes, particularly in the areas of health, employment, education and social services, in such a way that these services and programmes: Begin at the earliest possible stage, and are based on the multidiscip-linary assessment of individual needs and strengths; Support participation and inclusion in the community and all aspects of society, are voluntary, and are available to persons with disabilities as close as possible to their own communities, including in rural areas 2. States Parties shall promote the development of initial and continuing training for professionals and staff working in habilitation and rehabilitation services 3. States Parties shall promote the availability, knowledge and use of assistive devices and technologies, designed for persons with disabilities, as they relate to habilitation and rehabilitation
4. To ensure the **integrated and coordinated** provision of high-quality, affordable, accessible, gender-sensitive, appropriate, and **evidence-based interventions for rehabilitation along the continuum of care**	Improve health sector response to SCI Actions: Governments can improve provision of health, rehabilitation and support services for people with SCI; Service providers can help ensure a smooth transition between inpatient, outpatient and community-based care through establishment of a coordinated, integrated and multidisciplinary service approach; academia can increase the evidence base for interventions by fostering SCI research; promote access to specialist training to ensure an adequate supply of suitably trained health professionals	
5. To develop strong **multidisciplinary rehabilitation skills** suitable to the country context, including in all relevant health workers; to **strengthen capacity for analysis and prognosis** of workforce shortages	Improve health sector response to spinal cord injury Promote appropriate research and data collection Actions: promote access to specialist training to ensure an adequate supply of suitably trained health professionals; include SCI topics in the training curriculum for medical and allied health professionals to raise awareness about SCI and promote SCI research	
6. To enhance **health information systems to collect information relevant to rehabilitation**	Promote appropriate research and data collection Actions: Governments can promote standards for national SCI data collection, including centralized SCI registries; collect internationally comparable SCI information and make these data available in annual reports published on the Internet in a searchable manner so that data can be easily located	
7. To promote **high-quality rehabilitation research, including health policy and systems research**	Promote appropriate research and data collection Actions: Governments can promote standards for national SCI data collection, including centralized SCI registries; undertake research to determine the best possible rehabilitation measures to restore function in different contexts; collect internationally comparable SCI information and make these data available in annual reports published on the Internet in a searchable manner so that data can be easily located	
8. To ensure timely integration of rehabilitation into **emergency preparedness** and response, including emergency medical teams	Improve health sector response to spinal cord injury	**Article 11 – Situations of risk and humanitarian emergencies** States Parties shall take, in accordance with their obligations under international law, including international humanitarian law and international human rights law, all necessary measures to ensure the protection and safety of persons with disabilities in situations of risk, including situations of armed conflict, humanitarian emergencies, and the occurrence of natural disasters
9. To urge public and private stakeholders to stimulate investment in the development of available, affordable and usable **assistive technology**	Improve health sector response to SCI Actions: invest in the development of appropriate and affordable assistive technologies; ensure that products and services are accessible to people with disabilities, including people with SCI, in sectors such as health, sport, education	**Article 9 – Accessibility** 1. To enable persons with disabilities to live independently and participate fully in all aspects of life, States Parties shall take appropriate measures to ensure to persons with disabilities access, on an equal basis with others, to the physical environment, to transportation, to information and communications, including information and communications technologies and systems, and to other facilities and services open or provided to the public, both in urban and in rural areas. These measures, which shall include the identification and elimination of obstacles and barriers to accessibility, shall apply to, inter alia: Buildings, roads, transportation, and other indoor and outdoor facilities, including schools, housing, medical facilities and workplaces; Information, communications and other services, including electronic services and emergency services.

Overall and looking ahead, the 360° Model provides a robust foundation for advancing health research in a way that is not only scientifically rigorous but also socially responsible and ethically grounded. This programmatic vision calls for continued innovation and collaboration across disciplines, guided by a commitment to improving the quality of life for all individuals, regardless of the health challenges they face. The 360° Model of Research challenges us to rethink how we conduct health research, urging us to move beyond traditional silos and towards a future where research is fully integrated, inclusive, and impactful.
